# Case Report: The Added Value of Liquid Biopsy in Advanced Colorectal Cancer From Clinical Case Experiences

**DOI:** 10.3389/fphar.2021.745701

**Published:** 2021-11-10

**Authors:** Paola Ulivi, Alessandro Passardi, Giorgia Marisi, Elisa Chiadini, Chiara Molinari, Matteo Canale, Luigi Pasini, Fabio Ferroni, Giovanni Luca Frassineti, Giulia Bartolini, Manlio Monti

**Affiliations:** ^1^ Biosciences Laboratory, IRCCS Istituto Romagnolo per lo Studio dei Tumori (IRST) “Dino Amadori”, Meldola (FC), Italy; ^2^ Medical Oncology Unit, IRCCS Istituto Romagnolo per lo Studio dei Tumori (IRST) “Dino Amadori”, Meldola (FC), Italy; ^3^ Radiology Unit, IRCCS Istituto Romagnolo per lo Studio dei Tumori (IRST) “Dino Amadori”, Meldola (FC), Italy

**Keywords:** CtDNA, metastatic colorectal cancer, *RAS*, mutations, case report

## Abstract

Liquid biopsy represents a valid strategy for tumor molecular characterization. It gives the opportunity to bypass tumor heterogeneity, to monitor tumor characteristics during the course of treatment, and to perform the analysis even when tumor tissue is not available or inadequate. In the clinical practice of metastatic colorectal cancer, tumor molecular characterization is crucial for patient management, as *RAS* and *BRAF* status could influence the treatment choice. Although for this type of cancer tumor tissue is usually available at diagnosis, liquid biopsy could give complementary information and could permit monitoring of the mutation status during the course of treatment. At present, there are no clinical indications for its use in clinical practice. However, we report four clinical cases for which liquid biopsy analysis gave integrative information with respect to tumor tissue characterization, which permits us to understand the unresponsiveness of patients to treatment, with potential implications in patient’s management.

## Introduction

Accurate tumor molecular characterization is fundamental for targeted therapy and precision medicine. For advanced colorectal cancer (CRC), therapeutic agents against the epidermal growth factor receptor (EGFR) represent the standard of treatment for those patients carrying a wild type *RAS* status (Di Fiore et al., 2007; Karapetis et al., 2008). Usually, tumor tissue derived from surgery or endoscopic procedures represents the gold standard specimens for *RAS* molecular characterization. Contrary to other solid tumors, for which very often the tumor tissue available is very scarce and insufficient for the molecular determinations, for CRC the tumor material is usually enough to permit the performing of all the necessary molecular analyses. However, tumors are known to have spatial and temporal heterogeneity, and mutations not identified in primary tumors have been shown to be present in the metastatic lesions (Knijn et al., 2011; Furuki et al., 2018). Moreover, treatment could induce clonal evolution with the consequent acquisition of further molecular alteration (Misale et al., 2012; Yamada et al., 2016). Liquid biopsy, mainly in terms of circulating tumor DNA (ctDNA), represents a valid option to study the molecular characteristics of tumors, bypassing the issue of spatial heterogeneity and permitting the monitoring of the clonal evolution during treatment (Diehl et al., 2008; Murtaza et al., 2013). A recent study has demonstrated that the use of liquid biopsy could complement that of tumor tissue, showing that the combined use of the approaches could increase the diagnostic accuracy (Takeda et al., 2019).

To date, however, the use of liquid biopsy is not recommended in clinical practice for patients with advanced CRC, and it is performed only sporadically based on the Medical Oncologist’s request.

Here we present the history of four patients with advanced CRC, all with a baseline tumor tissue characterization revealing a *RAS* and *BRAF* wild type (wt) status and receiving an anti-EGFR treatment that was resistant to treatment. In all four patients, the liquid biopsy analysis revealed important information not evidenced by tumor tissue analysis.

## Case Presentations

### Case A

**FIGURE 1:**
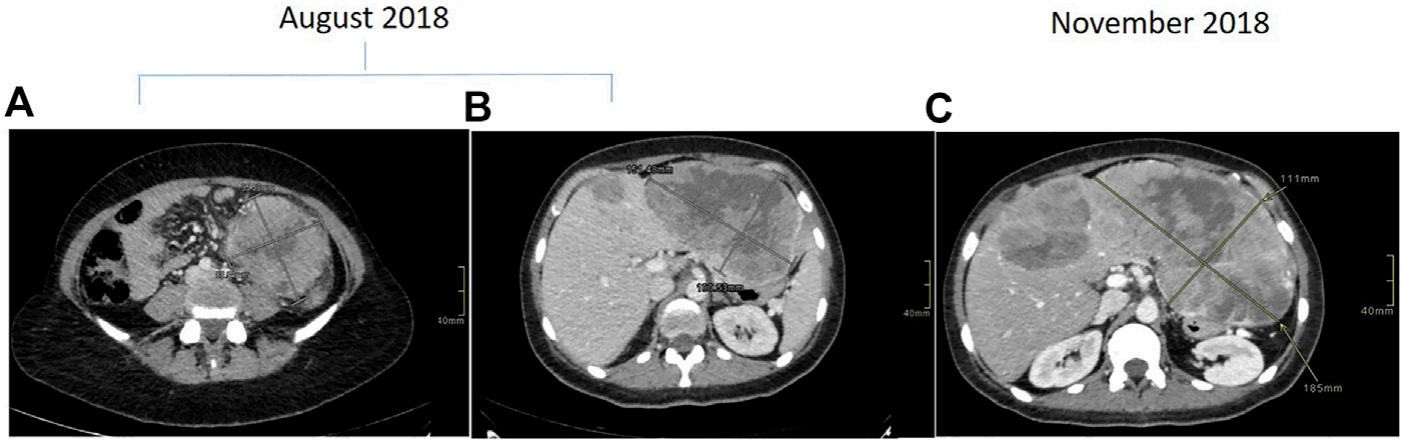
Contrast enhanced CT scan demonstrating descending colon cancer at baseline **(A)** and liver metastasis at baseline **(B**) and at progression **(C)**.

Towards mid-2018, a 40- to 45-year-old patient underwent colonoscopy because of persistent constipation, with a diagnosis of stenotic colorectal adenocarcinoma on splenic flexure. The chest and abdominal CT scan showed a metastatic disease, in particular a lesion on the left lobe of the liver of about 107 × 67 × 94 mm and six lesions on the right lobe, the bigger of which was about 21 mm (Figure 1A,B). There was also peritoneal carcinomatosis. At diagnosis CEA was 1.8 μg/L (<5 μg/L) and Ca 19–9 14.5 KU/L (<37 KU/L). The molecular characterization was performed on the primary tumor tissue using a MassARRAY Sequenom analysis (Myriapod Colon status, Diatech Pharmacogenetics, Jesi, Italy), revealing a *RAS* and *BRAF* wt status. Consequently, the patient received a first-line treatment with FOLFOXIRI (Irinotecan 150 mg/mq over 1 h day 1, oxaliplatin 85 mg/mq over 2 h day 1, calciolevofolinate 200 mg/mq over 2 h day 1, 5 fluorouracil 2400 mg/mq over 48 h) plus Panitumumab (6 mg/kg over 1 h day 1) every 2 weeks. After four cycles of treatment, CT scan evaluation showed disease progression, in particular on the left lobe of the liver where the bigger metastasis was 180 mm (Figure 1C), while there was an improvement on the primary tumor because the colorectal cancer step from 96 × 89 mm to 61 × 54 mm. Because of the young age of the patient, a liquid biopsy sample was made to obtain useful information for new drugs. *RAS* and *BRAF* status were analyzed by a Real-Time PCR method (Easy-KRAS status, Easy-NRAS status, Easy-BRAF status, Diatech Pharmacogenetics). Results showed the presence of a *BRAF* mutation (V600E). Concomitantly, the patient had a performance deterioration with severe abdominal pain and received a second-line treatment with FOLFIRI (Irinotecan 180 mg/mq over 90 min day 1, calciolevofolinate 200 mg/mq over 2 h day 1–2, 5 fluorouracil 400 mg/mq 3′-4′ day 1, 5 fluorouracil 2,400 mg/mq 48 h day 1) plus aflibercet (4 mg/kg over 1 h) every 2 weeks. Unfortunately, the patient rapidly progressed with a decline of performance status and died after 1 month from chemotherapy. Based on the results obtained on liquid biopsy about V600E *BRAF* mutation, the same analysis was repeated on DNA of the tumor tissue using a more sensitive methodology (Real-Time PCR), but a wt *BRAF* status was confirmed. These results suggest two hypotheses: 1) *BRAF* mutation was already present in the primary tumor tissue but heterogeneously, and tumor tissue analysis was not able to evidence the mutation; 2) *BRAF* mutation was induced during treatment as a secondary resistance mechanism.

### Case B

**FIGURE 2:**
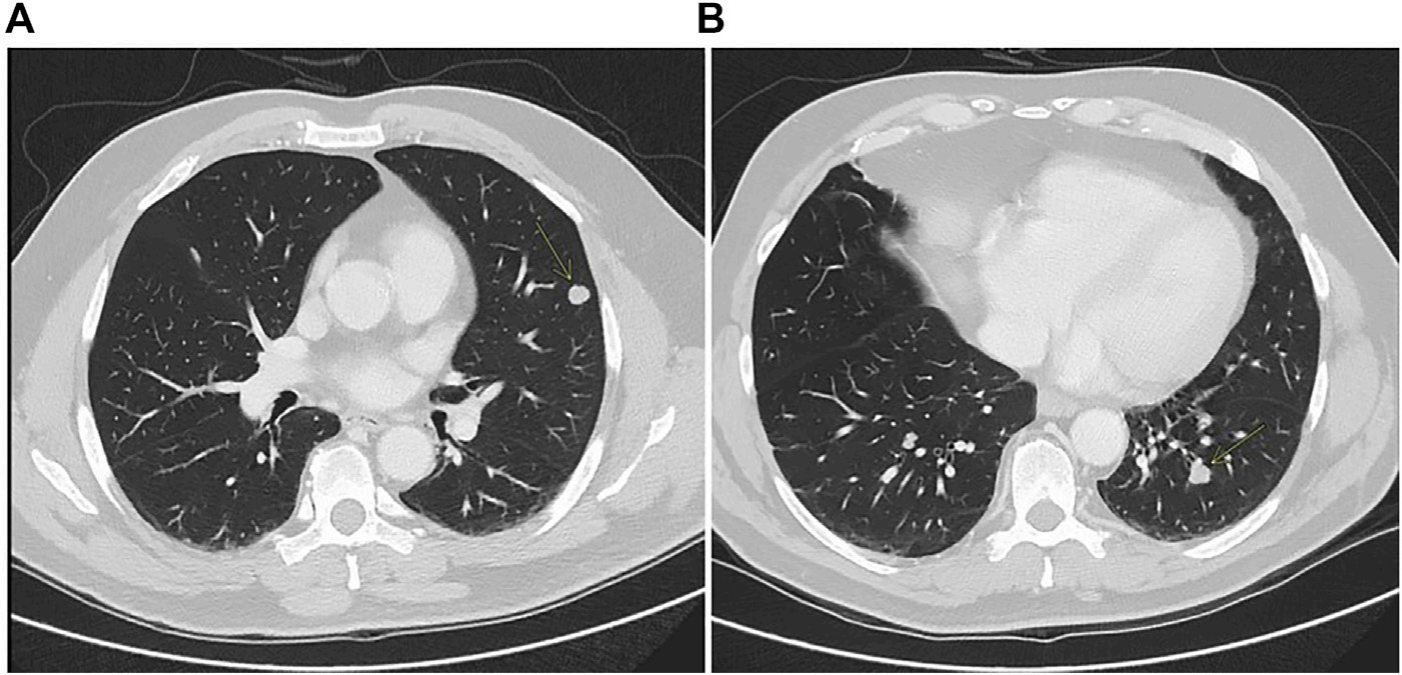
CT scan showing left lung metastases **(A,B)**.

At the end of 2014, a 55 to 60-year-old patient underwent pancolonscopy because of rectal bleeding, with a diagnosis of rectal adenocarcinoma placed at 8, 9 cm from anal rhyme. The abdominal and chest CT scan showed a localized disease on the proximal rectum. The pelvic NMR described a rectal cancer and the disease was a cT3N + M0. At diagnosis, CEA was 10.1 μg/L (<5 μg/L). The patient received a neo-adjuvant treatment inside a clinical trial, with FOLFOX4 (Oxaliplatin 85 mg/mq over 2 h day 1, calciolevofolinate 100 mg/mq over 2 h day 1–2, 5 fluorouracil 400 mg/mq 3′-4′ day 1–2, 5 fluorouracil 600 mg/mq 22 h day 1–2 every 2 weeks) for 4 cycles and hypofractionated radiotherapy (25 Gy/5 fr) that was administered between the second and the third cycle of chemotherapy, After the neoadjuvant treatment CEA was 5.7 µg/Land the patient underwent a surgical resection of rectum (ypT2N1cR0). After surgery, the patient performed an adjuvant therapy with FOLFOX4 for eight cycles and at the end of therapy, CEA was 4.8 μg/L. In early 2017 disease relapse was shown, with bilaterally pulmonary metastases at CT scan and CEA increased to 8.7 μg/L (Figure 2).

Molecular characterization performed using MassARRAY Sequenom (Myriapod Colon status, Diatech Pharmacogenetics) on the tumor tissue of the surgical resection of 2015 showed a *BRAF* and *RAS* wt status. Then the patient started a first-line treatment with FOLFIRI (Irinotecan 180 mg/mq over 90 min day 1, calciolevofolinate 200 mg/mq over 2 h day 1–2, 5 fluorouracil 400 mg/mq 3′-4′ day 1, 5 fluorouracil 2,400 mg/mq 48 h day 1 every 2 weeks) plus cetuximab (400 mg/mq over 2 h the first time and then 250 mg/mq over 1 h) for eight cycles with evidence of SD at CT scan and a slight increase of CEA that was 15.6 μg/L. Maintenance treatment with weekly cetuximab was then carried out but interrupted after only 2 months when a CT scan showed a progression disease on the lungs where the bigger lesion step from 12 × 10 to 15 × 14 mm on the inferior left lobe and CEA became 48.5 μg/L. Concomitantly we performed a liquid biopsy analysis and *RAS* and *BRAF* status were analyzed using a Real-Time PCR method (Easy-KRAS status, Easy-NRAS status, Easy-BRAF status, Diatech Pharmacogenetics). From this analysis, a *KRAS* G13D mutation was identified. In view of this result, *KRAS* status was determined in the primary tissue of 2015 using the Real-Time PCR assay, revealing a borderline *KRAS* G13D. These results highlight that the *KRAS* G13D was already present sub-clonally at the baseline, not identified using the standard mutation analysis methodology, and was responsible for the inefficacy of the anti-EGFR agent. After these evaluations, the patient received a second-line treatment with FOLFOX4 with an SD and improvement of CEA that went down to 10.8 μg/L after five cycles. After ten cycles of treatment an abdominal and chest CT scan showed a dimensional increase of most of the known lung lesions. The patient received Regorafenib (160 mg/die q 21) as third-line treatment for 9 months with stable disease as a better response but a progressive increase of CEA from 35 μg/L to 269 μg/L. At progression, Regorafenib was stopped and the patient started Trifluridine/Tipiracil (700 mg/mq q 28) for three cycles, with the radiological progression of disease and CEA increasing to 413 μg/L. The patient subsequently received metronomic capecitabine plus hyperthermia with further progression and was referred to palliative care. The patient died about 5 years after the first diagnosis of tumor.

### Case C

**FIGURE 3:**
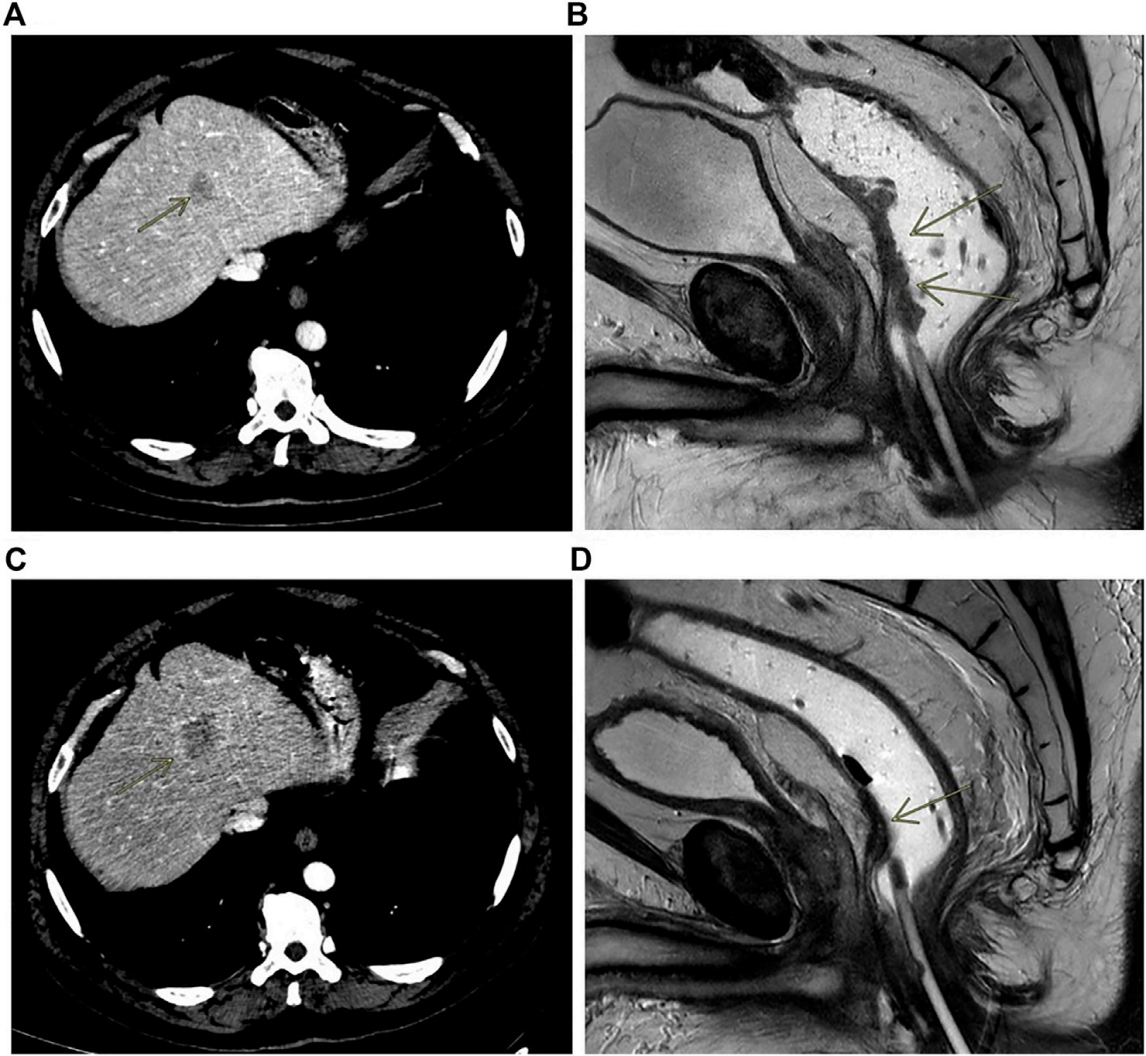
Contrast enhanced CT scan showing liver metastasis at baseline **(A)** and at progression **(C)**; MRI rectal cancer at baseline **(B)** and after treatment **(D)**.

In early 2018, a 55 to 60-years-old patient with persistent constipation and bleeding underwent colonoscopy, with a diagnosis of distal rectal adenocarcinoma with a single synchronous hepatic metastasis (about 25 mm) on IV–VIII segment (Figure 3A). CEA was 2.6 μg/L at diagnosis. A pelvic NMR described a rectal cancer extended from 2 cm above the anal opening to 10 cm, which was defined as cT3N1M1 (Figure 3B). Molecular characterization performed by MassARRAY Sequenom (Myriapod Cancer status, Diatech Pharmacogenetics) of the primary tissue revealed a *RAS* and *BRAF* wt status. The patient received a first-line treatment with FOLFOX6 (oxaliplatin 85 mg/mq over 2 h day 1, calciolefolinate 200 mg/mq over 2 h day 1, 5 fluorouracil over 3′-4′ day 1, 5 fluorouracil over2400 mg/mq over 48 h) plus panitumumab (6 mg/kg) every 2 weeks for five cycles and then capecitabine (825 mg/mq BID daily for 6 weeks) with concomitant radiotherapy. At the end of chemoradiotherapy, an abdominal and chest CT scan showed a progression of the liver disease on the IV-VIII segment from 24 × 20 mm to 42 × 36 mm (Figure 3C) while the pelvic NMR described a regression of the primary tumor (Figure 3D). The pathology report from an anterior resection of the rectum described a complete pathologic regression (pT0N0R0) of the primary tumor. It was not possible to perform the liver resection at the same time because of the appearance of a mixed acidosis that would have greatly prolonged the intervention. Therefore, we decided to restart treatment with FOLFOX6 and panitumumab. After 4 cycles, there was an increase of the liver disease up to 55 × 45 mm on IV-VIII segment, and the pathologic report from left hepatectomy reported intestinal adenocarcinoma with abundant necrosis. Molecular characterization of the metastasis performed by Next Generation Sequencing (NGS) (Oncomine Focus Assay, Thermofisher Scientific, Monza, Italy) revealed *NRAS* Q61K and *APC* c.4199C > A mutations. Liquid biopsy performed at the same time by Real-Time PCR (Easy-NRAS status, Diatech Pharmacogenetics) revealed the presence of *NRAS* Q61K mutation. These results revealed that in the metastatic lesion, a *NRAS* N61Q clone was selected and was responsible for the hepatic progression, and liquid biopsy was able to identify such mutation. The repetition of molecular analysis on DNA of the primary tissue, using a Real-Time PCR-based method, revealed a borderline presence of the *NRAS* Q61Q mutation. This result reinforced the hypothesis that the mutation was present sub-clonally in the primary tissue, but the low percentage of mutated cells did not obstacle the responsiveness to the anti-EGFR treatment. Conversely, the mutation was selected in the metastatic lesion, rendering it not sensitive to treatment. Liquid biopsy was able to identify the mutation.

An FDG-PET scan performed after liver surgery revealed an early liver and lung progression of disease, so a second-line treatment was started with FOLFIRI (Irinotecan 180 mg/mq over 90 min day 1, calciolevofolinate 200 mg/mq over 2 h day 1–2, 5 fluorouracil 400 mg/mq 3′-4′ day 1, 5 fluorouracil 2400 mg/mq 48 h day 1 every 2 weeks) plus bevacizumab (5 mg/kg over 90′ the first time, then 5 mg/kg over 1 h next) every 2 weeks After six cycles of treatment, CEA became positive until 7.2 μg/L and an abdominal and chest CT scan confirmed a progression of disease with multiple lung and liver metastases. Then the patient was treated with 4 cycles of Trifluridina/Tipiracil (700 mg/mq q 28) but there was again a progression of disease. The patient unfortunately died 2 months after.

### Case D

At the end of 2018, a 45- to 50-year-old patient underwent colonoscopy for abdominal pain with a diagnosis of adenocarcinoma on the right colon. The basal abdominal and chest CT scan described a liver metastasis on V, VI segment about 30 mm and a doubt lesion about 6 mm on the superior left lobe of the lung. At diagnosis CEA was 1.6 μg/L (<5 μg/L) and Ca 19–9 14.5 KU/L (<37 KU/L). The patient underwent right hemicolectomy and liver resection with a diagnosis of poorly differentiated carcinoma G3 of the colon, with focal aspects of squamous differentiation, and the presence of carcinomatous lymphangitis and hepatic metastasis from poorly differentiated carcinoma. Tissue molecular characterization was performed on specimens of the primary lesion in another center and revealed a *RAS* and *BRAF* wt status. Liquid biopsy analysis performed by a Real-Time PCR method (Easy-KRAS status, Easy-NRAS status, and Easy-BRAF status, Diatech Pharmacogenetics) confirmed a *RAS* and *BRAF* wt status.

**FIGURE 4:**
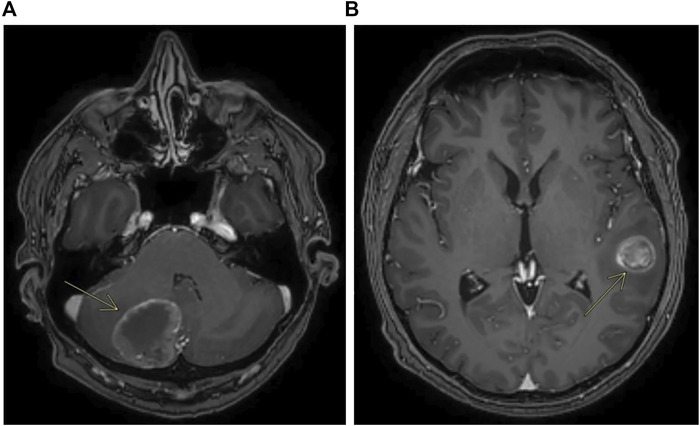
Contrast enhanced MRI showing metastases of the cerebellum **(A)** and metastases of the left temporal lobe **(B)**.

In early 2019, the patient received first-line chemotherapy with FOLFOX6 (oxaliplatin 85 mg/mq over 2 h day 1, calciolefolinate 200 mg/mq over 2 h day 1, 5 fluorouracil over 3′-4′ day 1, 5 fluorouracil over 2400 mg/mq over 48 h) plus panitumumab (6 mg/kg) every 2 weeks for five cycles, and the CT scan confirmed a lung metastatic lesion increased to 14 × 13 mm. An FDG-PET scan confirmed the single lesion. Then the patient was operated on in the superior left lobe of the lung, and the disease was about 15 mm. The diagnosis was poorly differentiated adenocarcinoma, with large necrotic areas compatible with metastases from carcinoma of the large intestine. Upon immunohistochemical investigation, neoplastic cells showed minimal expression of CDX2 and were negative for TTF1, neuroendocrine markers, and cytokeratins 7 and 20. The growth fraction was 70%. Molecular analysis on this lesion, made using an NGS Focus Oncomine assay (Thermofisher) revealed the presence of *PIK3CA* c.3073A > G and *MAP2K1* c.607G > A mutations. Liquid biopsy performed at the same time using the Oncomine Colon cfDNA Assay (Thermofisher Scientific) revealed the same *MAP2K1* mutations, together with an *APC* mutation (c.4463T > G) that was not covered by the Focus Oncomine assay performed on tissue. It was not possible to verify the presence of the *PIK3CA* mutation as that specific mutation was not analyzed in the Oncomine Colon cfDNA Assay. After surgery, post-operative chemotherapy with FOLFOX6 was administered. After 4 cycles of treatment an abdominal and chest CT scan did not describe metastatic disease, whereas a brain CT scan performed for referred headache, photophobia and dizziness unfortunately showed a cerebellar lesion about 35 mm on the right (Figure 4A) and another lesion about 20 × 20 mm on temporal left site (Figure 4B).

The patient received craniotomy with cerebellar right resection but the second lesion was not removed. The report was metastatic localization of poorly differentiated adenocarcinoma with large necrotic areas, compatible with known colic primitiveness. Upon immunohistochemical investigation, the cells were only focally positive for CDX2 and negative for CK20 and CK7, TTF1, and Chromogranin. The cell proliferation index, assessed by immunohistochemistry using Ki67/Mib1, was approximately 70%. Molecular analysis of this lesion, made by NGS Oncomine Focus assay, revealed the same spectrum of mutation found in the lung lesion (*PIK3CA* c.3073A > G, *MAP2K1* c.607G > A). At the end of 2019, mediastinal lymph nodes and liver metastases appeared on FDG-PET scan, in the same period the patient received brain tomotherapy (2,500 cGy 5 Fr). In early 2020, a second-line treatment with FOLFIRI (Irinotecan 180 mg/mq over 90 min day 1, calciolevofolinate 200 mg/mq over 2 h day 1–2, 5 fluorouracil 400 mg/mq 3′-4′ day 1, 5 fluorouracil 2400 mg/mq 48 h day 1 every 2 weeks) was administered for only two cycles but it was stopped because of grade 2 diarrhea and decline of performance status with clinical progression of disease.

By this case report, it has been highlighted that, in this case, liquid biopsy was able to identify the same spectrum of mutation found in tissue, permitting to evidence the presence of mutations carried by the metastatic lesions.

## Discussion

The four patients’ histories we reported highlighted the clinical potential utility of liquid biopsy analysis in the management of a patient with advanced CRC. In Case A, we reported a patient with a very aggressive left-sided colorectal tumor that was characterized as *RAS* and *BRAF* wt from the tumor molecular analysis. Liquid biopsy analysis performed a few months later revealed the presence of a *BRAF* V600E mutation in accordance with the highly aggressive tumor behavior (Sanz-Garcia et al., 2017). Considering that the repetition of the analysis on the primary tissue using a more sensitive methodology was not able to reveal the mutation, probably that mutation was present in a heterogeneous manner, unable to be evidenced by a tissue analysis performed on a specific tumor site. Instead, liquid biopsy was able to evidence the mutation, bypassing the problem of heterogeneity. In this case, the knowledge from the beginning of the *BRAF* V600E mutation would have permitted a different treatment, like FOLFOXIRI and bevacizumab, and in second-line, Binimetinib, Encorafeninb and Cetuximab.

A similar advantage of liquid biopsy was seen for Cases B and C, where the *RAS* mutation was evidenced by liquid biopsy and not by tumor tissue analysis with standard methodology. In both cases, the repetition of the analysis on tumor tissue with a more sensitive methodology revealed a borderline presence of the mutation. These findings again highlighted the heterogeneous presence of the mutation from the beginning, evidenced by liquid biopsy but not by tumor tissue determination. The *KRAS* G13D mutation in Case B was the cause of the very low responsiveness to anti-EGFR therapy, and knowledge of the mutation status from the beginning would have indicated an alternative treatment for the patient. Similarly, the *NRAS* N61Q mutation in Case C was evident in the metastatic lesion at relapse and in the liquid biopsy, but was present at borderline and evidenced only using very sensitive methodologies in the primary tumor tissue. In addition, in this case, the knowledge of the mutation would have enabled treating the patient with an alternative treatment strategy. Probably we would have used anti-VEGF in association to chemotherapy upfront and over progression.

The last case we reported (Case D) highlighted that gene alterations other than *RAS* and *BRAF* could be responsible for the unresponsiveness to anti-EGFR agents. In fact, *PIK3CA* and *MAP2K1* mutations were seen in the metastatic lesions and liquid biopsy. Although it was not possible to verify the presence of such mutations from the beginning, as a targeted sequencing was performed instead of an NGS approach, we observed that the mutation was determinable in liquid biopsy.

Different methodologies are used for *RAS* and *BRAF* determinations, having different sensitivity of detection ranging from 0.1 to 5% (Tan and Du, 2012; Taniguchi et al., 2015). Many molecular diagnostic laboratories do not routinely use ultrasensitive methodologies for tumor tissue molecular characterization, and some under-represented mutations could be missed. This is true, in particular, if the mutation is heterogeneously present within the tumor (Gargalionis and Papavassiliou, 2017; Khan et al., 2018; Molinari et al., 2018). Liquid biopsy analysis could represent an optimal option, representing the overall tumor spectrum mutation.

Another important advantage of liquid biopsy is the possibility to monitor and identify mutations that will arise from the primary tumor to the metastatic lesion (Siravegna et al., 2015). Several reports have demonstrated the possibility to monitor, through liquid biopsy, the arising of resistance mutations during treatment with anti-EGFR therapies (Gargalionis and Papavassiliou, 2017; Khan et al., 2018; Chen et al., 2019; Kastrisiou et al., 2019).

We have demonstrated that NGS analysis on liquid biopsies could be feasible and could permit us to analyze concomitantly different gene mutations, permitting us to evidence new alterations arising during treatment that could potentially be the target of subsequent targeted treatments. A recent study showed that liquid biopsy analysis allowed an increase in therapeutic options in one third of analyzed patients (Pereira et al., 2020). Moreover, clinical trials have been performed in which liquid biopsy was used to drive clinical decisions. In a phase II trial designed by Cremolini et al. it was demonstrated that a challenging strategy with cetuximab and irinotecan may be active in patients with *RAS* and *BRAF* wt mCRC with acquired resistance to first-line irinotecan- and cetuximab-based therapy. The evaluation of *RAS* mutational status on ctDNA might be helpful in selecting candidate patients.

The increasing consolidation of the use of NGS methodologies in liquid biopsy has opened the possibility to monitor multiple gene alterations, with potentialities in monitoring response to treatment and in identifying emerging resistance mutations (Kastrisiou et al., 2019).

Despite the potential of NGS on liquid biopsy in mCRC, the specific limitations have to be considered. The principal limitation is sensitivity. The issue of sensitivity is highly relevant in the context of liquid biopsies, where mutant ctDNA is like a “needle in a haystack” of wt DNA fragments. Some other high sensitive methodologies have been proposed for evaluation of mutations in liquid biopsies, such as digital droplet PCR (Ono et al., 2017; Decraene et al., 2018) also applied to extracellular vesicles (Notarangelo et al., 2019). However, the increasing knowledge of tumor mutations and the increase of targeted therapeutic drugs available render NGS analysis more suitable for a wide molecular characterization. In the future, the development of NGS platforms with higher sensitivity will further upgrade the study of tumor heterogeneity in the blood. The use of targeted NGS to monitor these mutations in real-time may set the foundation for a new approach in the management of cancer.

## Conclusion

By these four clinical case reports we highlighted that liquid biopsy could have a role in the management of mCRC patients, giving complementary information that could be very important for the treatment decision making. Tumor heterogeneity and its dynamic evolution over time are both aspects that could affect the reliability of tumor tissue analysis. Although no indications are present for the use of liquid biopsy in the clinical management of mCRC patients, clinicians could consider it as an option in specific circumstances. The test is rapid and with a relatively low cost, both aspects that facilitate its use in the clinical practice. There is currently no indication to administer targeted drugs (outside of clinical trials) based on mutations identified by liquid biopsy, but it is desirable that this can happen in the near future to expand the therapeutic opportunities of our patients.

## Data Availability

The datasets presented in this article are not readily available due to ethical and privacy restrictions. Requests to access the datasets should be directed to the corresponding author.
